# Population genetic analysis of bi-allelic structural variants from low-coverage sequence data with an expectation-maximization algorithm

**DOI:** 10.1186/1471-2105-15-163

**Published:** 2014-05-29

**Authors:** José Ignacio Lucas-Lledó, David Vicente-Salvador, Cristina Aguado, Mario Cáceres

**Affiliations:** 1 Institut de Biotecnologia i de Biomedicina, Universitat Autònoma de Barcelona, 08193 Bellaterra (Barcelona), Spain; 2 Leibniz-Institute of Freshwater Ecology and Inland Fisheries (IGB), 12587 Berlin, Germany; 3 Institució Catalana de Recerca i Estudis Avançats (ICREA), 08010 Barcelona, Spain

**Keywords:** Structural variation, Population genetics, Maximum likelihood, Reference bias, Genotyping

## Abstract

**Background:**

Population genetics and association studies usually rely on a set of known variable sites that are then genotyped in subsequent samples, because it is easier to genotype than to discover the variation. This is also true for structural variation detected from sequence data. However, the genotypes at known variable sites can only be inferred with uncertainty from low coverage data. Thus, statistical approaches that infer genotype likelihoods, test hypotheses, and estimate population parameters without requiring accurate genotypes are becoming popular. Unfortunately, the current implementations of these methods are intended to analyse only single nucleotide and short indel variation, and they usually assume that the two alleles in a heterozygous individual are sampled with equal probability. This is generally false for structural variants detected with paired ends or split reads. Therefore, the population genetics of structural variants cannot be studied, unless a painstaking and potentially biased genotyping is performed first.

**Results:**

We present *svgem*, an expectation-maximization implementation to estimate allele and genotype frequencies, calculate genotype posterior probabilities, and test for Hardy-Weinberg equilibrium and for population differences, from the numbers of times the alleles are observed in each individual. Although applicable to single nucleotide variation, it aims at bi-allelic structural variation of any type, observed by either split reads or paired ends, with arbitrarily high allele sampling bias. We test *svgem* with simulated and real data from the 1000 Genomes Project.

**Conclusions:**

*svgem* makes it possible to use low-coverage sequencing data to study the population distribution of structural variants without having to know their genotypes. Furthermore, this advance allows the combined analysis of structural and nucleotide variation within the same genotype-free statistical framework, thus preventing biases introduced by genotype imputation.

## Background

Ongoing efforts to discover genetic variation in humans and other species are yielding long lists of known variants [1-3]. The discovery of genetic variation is always the first step of population genetics or association studies. Once structural or nucleotide variation is revealed, individuals not present in the original sample are usually genotyped, for subsequent studies. Genotyping individuals is much easier than discovering new variants, because fewer loci have to be tested, and the prior probability of there being an alternative allele is higher than in sites not known to be variable. However, the presence of sequencing and mapping errors, and undersampling at heterozygous sites demand high coverage in order to infer individual genotypes accurately. Accurate genotypes are the basic information upon which most classic methods of population genetics depend. Our reliance on classic methods and the convenience of low-coverage data are such that the population-level structure of the variation is being used to improve the genotype calls on low-coverage data [4,5], which are then expected to help us understand the population-level structure of the variation. This circularity prevents, for example, the use of genotypes imputed on the bases of known patterns of linkage disequilibrium to infer recombination rates. The problem stems from considering the genotypes as the ultimate result of a research program. For most studies, other than personalized medicine, the genotypes are just the means to gain insight into population-level processes: association between genotypes and phenotypes, history of migration and admixture, patterns of recombination, natural selection, etc.

When studying structural variants, there is too much to loose from relying on imputed genotypes. Polymorphic structural variants contribute to phenotypic diversity and disease susceptibility in humans [6], and other species [7-11]. The population genetics of structural variants is both a classic field, and, thanks to the new sequencing techniques, an emergent research venue [12,13]. In the last years, many programs have been developed to identify structural variants from sequence data, using diverse signatures from split reads [14,15], read depth [16,17], paired reads [18,19], or a combination thereof [20,21]. Among the hotest topics that await to harvest full benefits from the high-throughput sequencing technologies, is the interplay between structural and nucleotide variation. The nucleotide variation linked to structural variants can inform of their evolutionary history and their effects on fitness, and it can also reveal to what extent structural variants affect recombination patterns. However, these questions cannot be addressed with SNP genotypes imputed on the bases of assumed patterns of linkage disequilibrium.

An alternative to genotype imputation is to study the population-level structure of the variation with new methods that take genotype uncertainty into account in the analysis. This is what has been proposed before for single-nucleotide variation [22-24]. The idea is to calculate the genotype likelihoods at every site, instead of calling the genotypes. Genotype likelihoods can be used to obtain unbiased estimates of allele frequencies or site-frequency spectra, and likelihood-ratio tests can be used to address population-level hypotheses, such as Hardy-Weinberg equilibrium, population differentiation, linkage disequilibrium, genotype-phenotype association, etc [24,25]. However, this promissing approach is not applicable yet to structural variants, mostly because existing implementations assume even sampling probabilities of the two alleles from a heterozygous sample. While this assumption is a reasonable simplification for the analysis of SNP data, it is not for the analysis of structural variants, where one of the alleles may be observed much more frequently than the other in a heterozygous sample [26]. One source of bias is the conservative alignment of reads to the reference genome. By default, mapping tools are optimized for the discovery phase, when the prior of there being a structural variant is very low. Therefore, aligners favor concordant mappings, that is, those that do not report the variation [27-29]. Another source of allele sampling bias is the different detectability of the alleles. For example, using single reads, the presence of a polymorphic insertion may be revealed by any read mapping to either of the two breakpoints, or even to its inner sequence, if unique and known; while the absence of the inserted fragment can only be positively attested by reads mapping on a single breakpoint. Also, repetitive sequences are frequently present around a structural variant, and they can impair the detection of one allele more than the other. For example, when the sequence of a transposable element is broken by an inversion in one of the breakpoints. In summary, allele sampling bias is the rule, rather than the exception, when genotyping structural variation with sequence data; and the bias can be orders of magnitude higher than in the case of SNPs (see below). In addition, the few existing tools able to report genotype likelihoods [5,24] require a bam file [30] for their calculation, which is not designed for structural variation at all.

Here, we present *svgem*, a simple and flexible command-line application to infer genotype likelihoods and allele frequencies from counts of reference and alternative alleles, appropriate for structural variation data, with an arbitrarily high reference (or alternative) bias. This program is not concerned with the discovery phase of the structural variants, but with the post-discovery analyses. The only assumptions made about the type of variation that can be analysed are that it is bi-allelic, and that the two alleles can be distinguished from sequencing data. Thus, simple insertions, deletions, inversions, and translocations of any length can be analysed with *svgem*, while multiple, overlapping rearrangements, with more than two possible alleles are not. The sampling bias must be known in advance, and passed as a parameter. We offer some guidelines on how to estimate it, and explain how to test for Hardy-Weinberg equilibrium. Also, the ploidy of the samples is taken into account to infer genotype likelihoods and allele frequencies in sex chromosomes properly.

## Implementation

### Overview and requirements

The program *svgem* implements an expectation-maximization algorithm in C++. The source code is freely available in Additional file 1, and in [31]. It runs from the command line, analyses one structural variant at a time, and it takes as input a text file with three (optionally, four) columns: a sample identifier and the numbers of times the reference and the alternative alleles have been observed. The optional fourth column represents the ploidy, for the case when the sample is composed of a mixture of males and females, and the variant being analysed is on the sex chromosome.

In order to make *svgem* compatible with virtually any way of counting the observations of reference and alternative alleles, only two qualities are distinguished: the average quality of the reference counts, and the average quality of the alternative counts. These are analogous to the base qualities of a sequenced read, and they can be passed as parameters to the program, in terms of the frequencies of erroneous counts, or estimated from the data. Alternatively, if the individual quality (probability of error) of each observation is known, the expected number of true counts or ‘effective’ number of times the alleles are observed, instead of the raw counts, can be used. This approach proved to be very useful in SNPTools [5].

The allele sampling bias is represented by one parameter, *λ*, defined as the odds of sampling the reference allele from a diploid, heterozygous genotype. Even though in some cases it could be estimated from the data, *svgem* requires *λ* to be passed as a parameter, in order to save degrees of freedom. Otherwise, it would be impossible to get accurate estimates of extreme allele frequencies in the presence of errors. Plus, because *svgem* is not designed to discover variants, but to analyse already known variants, it is fairly easy to estimate *λ*. There are two different situations. First, if the exact sequences of both structural alleles are completely known, the alleles can be distinguished by the single reads mapping specifically to one of them. All possible reads from the informative regions of both alleles can be extracted, as if they came from a heterozygous sample, and mapped back to the reference genome and to the alternative allele. Then, *λ* would be estimated as the ratio between the number of reference reads that map uniquely to the reference allele, and the number of alternative reads that map uniquely to the alternative allele.

Second, if the exact sequences of the structural alleles are not known (e.g., imprecise breakpoints), they must be distinguished by the pattern of paired-end mappings: concordant for the reference, and discordant for the alternative (methods based on the depth of coverage, usually requiring high coverage, are not considered here). Still, the most likely version of the alternative allele could be composed, and used to extract paired end reads from it. In this case, the complete extraction of all possible paired-end reads is not feasible, but extensive simulations can be done with available programs, such as *wgsim*[24], or *ART*[32]. If the same coverage is simulated in both alleles, *λ* can be estimated as the ratio of the number of reference paired ends that map concordantly to the number of alternative paired ends that map discordantly. To overcome the imprecision of the breakpoints, several alternative alleles could be simulated and averaged. Alternatively, if a subset of individuals are known to be heterozygous by other means (e.g., PCR evidence, or higher sequencing coverage), the ratio between their pooled numbers of reference and alternative reads can also be used as an estimate of *λ*. Inaccurate estimates of *λ* have a mild impact on genotype likelihoods, and they are always preferred to the default value of *λ*=1, as long as they are closer to the true value of the allele sampling bias (see below).

### Implementation of the expectation-maximization algorithm

Following the notation in [24] (see Table 1), we refer to a genotype by its number of reference alleles, *g* ∈ {0, 1 … *m*}, where *m* is the ploidy, usually 2. We assume that variants are biallelic, so that *m* - *g* is the number of alternative alleles in the genotype. Table 2 shows the likelihoods of the three diploid genotypes. The main difference with respect to Li’s equation 2 [24] is the heterozygous genotype, the likelihood of which depends here on the allele sampling bias. If *λ* = 1, and allowing for all the observations of the same allele to have the same quality, the difference vanishes (see Additional file 2). The likelihoods of the hemizygous genotypes Alt/0 and Ref/0 are the same as those of the respective homozygous genotypes.

**Table 1:** Notation

*k*	Total number of allele observations, or counts, in one individual.
*l*	Number of times the reference allele is observed in one individual (*l* ≤ *k*).
*m*	Ploidy.
*g*	Number of reference alleles in the genotype (*g* ≤ *m*).
*λ*	Allele sampling bias in heterozygous individuals.
*ε*_*r*_	Frequency of erroneous counts among reference counts.
*ε*_*a*_	Frequency of erroneous counts among alternative counts.

**Table 2:** **Likelihoods of the three diploid genotypes (*m = *** **2)**

**Genotype (*g*)**	**Likelihood**
0	$\varepsilon_{r}^{l}{\left(1-\varepsilon_{a}\right)}^{k-l}$
1	${\left(\frac{1}{1+\lambda }\right)}^{k}{\left(\varepsilon_{r}+\lambda -\lambda \varepsilon_{r}\right)}^{l}{\left(1-\varepsilon_{a}+\lambda \varepsilon_{a}\right)}^{k-l}$
2	${\left(1-\varepsilon_{r}\right)}^{l}\varepsilon_{a}^{k-l}$

Treating the genotypes as missing values, we implement an expectation-maximization (EM) method to estimate either the alternative allele frequency, *ψ*, under the assumption of Hardy-Weinberg equilibrium, or the genotype frequencies *ψ*_*g*_ (with *g* ∈ {0, 1, 2} for diploids) or *ϕ*_*g*_ (with *g* ∈ {0, 1}, for hemizygous individuals), and eventually the proportions of errors among reference (*ε*_*r*_) and alternative (*ε*_*a*_) counts. Note that *ψ*_*g*_ is the frequency of genotype *g* among diploids, and *ϕ*_*g*_ is the frequency of genotype *g* among hemizygous individuals. The EM algorithm is an iterative estimation of the parameters that gets closer to the maximum likelihood estimates in every iteration. Additional file 2 gives a summary of how the standard formulation of the EM algorithm is used to derive the next values of these parameters, namely:

$$\begin{array}{lll} \psi^{\left(t+1\right)} & =\frac{2D_{0}^{\left(t\right)}+D_{1}^{\left(t\right)}+H_{0}^{\left(t\right)}}{2(D_{2}^{\left(t\right)}+D_{1}^{\left(t\right)}+D_{0}^{\left(t\right)})+H_{0}^{\left(t\right)}+H_{1}^{\left(t\right)}}\; & \;\\ \psi_{g}^{\left(t+1\right)} & =\frac{D_{g}^{\left(t\right)}}{D}\; & \;\\ \phi_{g}^{\left(t+1\right)} & =\frac{H_{g}^{\left(t\right)}}{H}\; & \;\\ \varepsilon_{a}^{\left(t+1\right)} & =\frac{A_{2}^{\left(t\right)}}{A_{0}^{\left(t\right)}+A_{2}^{\left(t\right)}},\;\text{if}\;\lambda =1\; & \;\\ \varepsilon_{r}^{\left(t+1\right)} & =\frac{R_{0}^{\left(t\right)}}{R_{0}^{\left(t\right)}+R_{2}^{\left(t\right)}},\;\text{if}\;\lambda =1\; & \; \end{array}$$

In the equations above, $D_{g}^{\left(t\right)}$ is the *t*^*th*^ estimate of the total number of diploid individuals with genotype *g*, and $H_{g}^{\left(t\right)}$ is the *t*^*th*^ estimate of the total number of hemizygous individuals with genotype *g*. That is, they are the summations of the posterior probabilities of genotype *g* over the respective kind of individuals. *D* and *H* are the total number of diploid and hemizygous individuals, respectively, where *D* + *H* = *N*. $A_{g}^{\left(t\right)}$ is the *t*^*th*^ estimate of the total number of alternative counts coming from hemizygous and homozygous individuals for either the alternative (*g* = 0) or the reference (*g* = 2) allele. Finally, $R_{g}^{\left(t\right)}$ is the *t*^*th*^ estimate of the total number of reference counts that come from hemizygous and homozygous individuals for either the alternative (*g* = 0) or the reference (*g* = 2) allele.

When there is sampling bias in heterozygous individuals, *λ*≠1, and the next values of the proportions of errors among reference (*ε*_*r*_) and alternative (*ε*_*a*_) counts are the results of two quadratic equations (Additional file 2). In practice, it is assumed that the erroneous counts are a minority, and the program halts when *ε*_*r*_ ≥ 0.5 or *ε*_*a*_ ≥ 0.5. This can prevent the correct estimation of extreme allele frequencies in the presence of erroneous counts, as should be expected.

### Output and applications

The output includes: maximum likelihood estimates of the parameters mentioned above, the likelihood of such estimates, the genotype likelihoods of all individuals, and the posterior probabilities of the genotypes of all individuals. The main purpose of *svgem* is to obtain unbiased estimates of allele and genotype frequencies, which are fundamental parameters in population genetics. From these estimates, several other population parameters can be estimated. The maximum likelihood estimate of the frequency of the heterozygous genotype (${\hat{\psi }}_{1}$), estimated without assuming Hardy-Weinberg equilibrium, is a direct estimate of heterozygosity. An estimate of the inbreeding coefficient follows from comparing ${\hat{\psi }}_{1}$ with the expected frequency of heterozygous individuals under Hardy-Weinberg equilibrium: $\hat{F}=1-{\hat{\psi }}_{1}/(2\hat{\psi }(1-\hat{\psi }))$ (where $\hat{\psi }$ is the maximum likelihood estimate of the alternative allele frequency). The fixation index, *F*_ST_, which measures genetic differentiation among populations, is also readily estimated from allele frequencies [33-35]. A test for Hardy-Weinberg equilibrium (HWE) can be performed by running *svgem* with and without the equilibrium assumption, and comparing the log-likelihoods of the estimated frequencies. Twice the difference between the log-likelihoods must be compared with a *χ*^2^ distribution with 1 degree of freedom, if all individuals are diploid, or with 2 degrees of freedom if the frequencies of hemizygous genotypes are also being estimated.

Some analyses that used to require accurate knowledge of individual genotypes can be performed now using only genotype likelihoods. For example, it is possible to estimate the linkage disequilibrium between pairs of variants using genotype likelihoods, instead of individual genotypes [24]. At the end of the next section, we show how to estimate the linkage disequilibrium between a structural variant and the SNPs around it, without the biases typically associated with genotype imputation.

It is also possible to run genetic association tests from genotype likelihoods, without knowing the exact genotype of the individuals [24]. Associations between phenotypes and genetic variants are a significant difference in allele frequency between two samples (cases and controls), and they are routinely searched along the human genome to infer the causal variants of diseases. To compare the allele frequency of a variant between two samples, *svgem* must be run three times: once in each sample separately, and once in the joint dataset. Lets call *ℓ*_*a*_ and *ℓ*_*b*_ the log-likelihoods of the two independent estimates for samples *a* and *b*. The total log-likelihood of the hypothesis of two different frequencies is just the sum of the log-likelihoods of the two samples: *ℓ*_1_ = *ℓ*_*a*_ + *ℓ*_*b*_. The log-likelihood of the hypothesis of one common allele frequency, *ℓ*_0_, is obtained from the run on the joint data set. Because the two hypotheses are nested, the application of a likelihood ratio test is justified. Thus, if the null hypothesis of a common allele frequency is true, the statistic 2(*ℓ*_1_ - *ℓ*_0_) is expected to follow a *χ*^2^ distribution with as many degrees of freedom as additional parameters the most complex model has, which is 1 in this case (see [36], page 137).

For other analyses, that may require knowledge of individual genotypes, we recommend using the genotype with the highest posterior probability, which is more accurate than the most likely genotype, because posterior probabilities take into account the information of the genotype frequency in the population (see below). *svgem* uses the maximum likelihood estimates of allele ($\hat{\psi }$) or genotype (${\hat{\psi }}_{g}$) frequencies, and the genotype likelihoods (ℒ(g)) to calculate the genotype posterior probabilities, *P*(*g*∣data):

$$\;P(g|\text{data})=\left\{\begin{array}{ll} \frac{ℒ(g)P(g|\hat{\psi })}{\overset{m}{\underset{g=0}{\sum }}P(g|\hat{\psi })ℒ(g)} & \;\;\text{if HWE is assumed}\\ \frac{ℒ(g){\hat{\psi }}_{g}}{\overset{m}{\underset{g=0}{\sum }}{\hat{\psi }}_{g}ℒ(g)} & \;\;\text{if it is not} \end{array}\right)$$

### Performance

Although *svgem* is designed to analyse one variant at a time, more than one variant can easily be analysed in multiple parallel runs. For the purpose of detailed population genetic analyses of specific variants of interest, *svgem* performs well, since its typical run time is always a tiny fraction of the time usually needed to obtain the allele counts. In absolute terms, it can analyse >1000 individuals in, at most, a few seconds, in a standard PC. However, the run time is variable, just as in any expectation-maximization algorithm, and convergence may take longer if the information content of the input data set is limited.

## Results and discussion

### Estimates of ***λ*** from real structural variants

In order to assess the expected range of values of the *λ* parameter, we downloaded the BreakDB database [37], last accessed on January 26th 2014, and built a library with the known sequences of both alleles of 568 structural variants (54 inversions, 161 insertions, and 353 deletions). Then, we simulated the exhaustive sequencing of both alleles with single-end, 100-bp reads. Next, we mapped the sequenced reads first to the library built with the sequences of the reference and the alternative alleles, and afterwards to the whole reference genome (HG18), in order to discard any non-specific read. For this purpose, we used the pipeline BreakSeq [38], with minor modifications. We removed from BreakSeq a filter that required the reads to map on the breakpoints, in order to be able to use the inserted or deleted sequences as evidence of the presence or the absence of an insertion or a deletion. Finally, we counted how many reads mapped specifically on the reference or on the alternative allele, and estimated *λ* as the ratio of the two counts.

**Figure 1:**
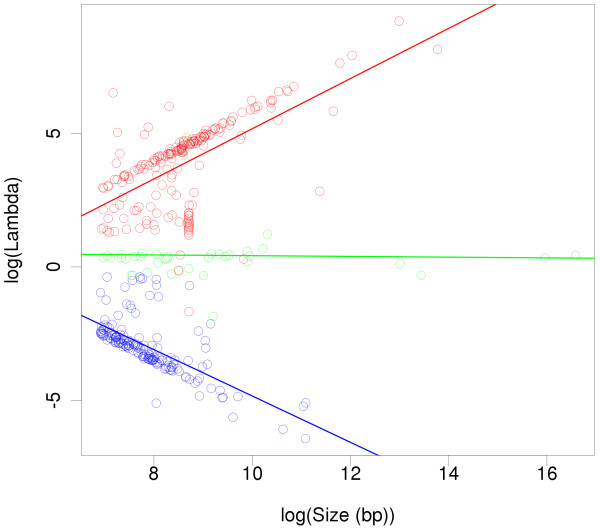
**Estimates of the allele sampling bias, *λ*, of real variants.** The allele sampling bias of 195 deletions (red), 43 inversions (green), and 161 insertions (blue) is plotted against their lengths, in logarithmic scales. The bias, *λ*, was estimated by simulating the sequencing of both alleles with 100 bp, single-end reads. Lines are linear regressions.

The observed, finite values of *λ* ranged between 0.002 and 10000 (11 inversions, and 158 deletions had infinite *λ*, meaning that one of the alleles was not detectable by sequencing with single-end, 100 bp reads, due to the presence of repeats around the breakpoints). Figure 1 shows that the length of the insertions and deletions greatly contributes to the value of *λ*, as expected. Because the inserted or deleted sequence is known, and used as evidence of the presence of the longest allele (the reference in a deletion, the alternative in an insertion), longer insertions or deletions produce more unbalanced allele observations, favouring the reference allele in a deletion (high *λ*) or the alternative one in an insertion (low *λ*). The local sequence around and within the variant, and the method used to detect the alleles (read length, whether single or paired-end) must also influence the exact value of *λ*. However, the linear regressions between the logarithm of *λ* and the logarithm of the size of the insertion or deletion (in base pairs) allow for a rough, first approximation to *λ*, at least when detecting insertions or deletions with single-end sequenced reads: log(*λ*) = -4.22 + 0.94 log(size) for deletions with respect to the reference allele (adjusted *R*^2^ = 0.37), and log(*λ*) = 3.84 - 0.87 log(size) for insertions (adjusted *R*^2^ = 0.44). In the case of inversions, a *λ* = 1 is a fair assumption, in the absence of additional information. This approximations are not expected to hold when detecting structural variants flanked by segmental duplications or other repeats.

### Analysis of simulated data

We run some simulations to test *svgem*. In the artificial datasets, genotypes followed Hardy-Weinberg equilibrium, with allele frequencies between 0.01 and 0.99, and the allele counts were sampled with a simulated error rate of 0.005. Coverage was Poisson-distributed, in order to introduce variation in coverage among individuals, although the exact distribution is irrelevant for the calculation of genotype likelihoods. In all, 100 simulations were run with the same parameter values.

**Figure 2:**
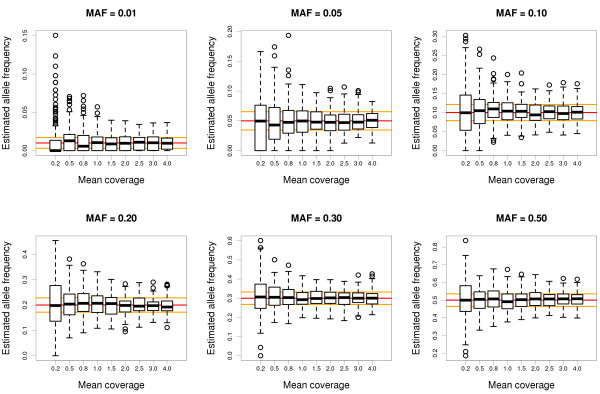
**Accuracy and precision of allele frequency estimates with low-coverage data.** Estimates obtained by *svgem* from random samples of 100 diploid individuals, from populations with alternative allele frequencies between 0.01 and 0.5 (upper label of each plot; MAF, minor allele frequency), for mean coverage depths between 0.2 and 4. Each combination of allele frequency and depth of coverage was simulated 100 times. The red line indicates the true allele frequency, and the orange lines indicate the expected standard error of the true frequency, from a sample of 200 chromosomes.

**Figure 3:**
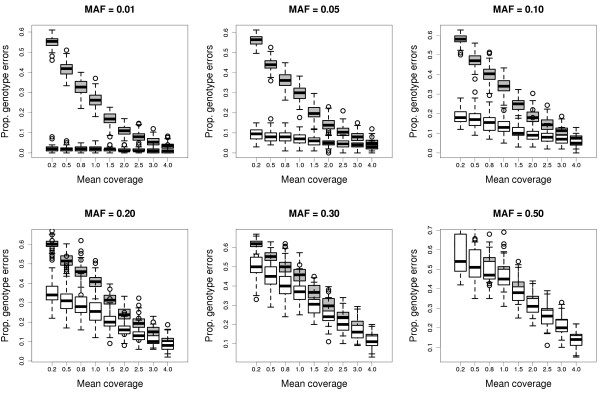
**Accuracy of genotype calls with low-coverage data.** Proportion of most likely (grey) or most probable (white) genotypes that do not match the true genotype among 100 individuals, in a series of simulations with different values of allele frequency and coverage. The minor allele frequencies (MAF) are shown above each plot. Each box corresponds to 100 simulations.

First, we checked that *svgem* is able to get unbiased estimates of the allele frequency with low coverage data, in the absence of any allele sampling bias (*λ* = 1). Figure 2 shows how estimates based on a sample of 100 individuals are accurate and as precise as they can be with mean coverages ranging from 0.2 to 4. The only biased estimates correspond to alternative allele frequencies lower than 0.01 (or higher than 0.99, not shown), targeted with sequencing coverages lower than 0.5. Not surprisingly, as the number of parameters to estimate increases, the precision and the accuracy drop. The frequencies of the three genotypes can still be estimated without bias in most cases, if the mean coverage is higher than 2 (Figures S2–S4 in Additional file 2). When comparing the true genotypes of all individuals simulated with the most likely and with the most probable genotypes (Figure 3), two results become aparent: 1) the benefit of using posterior probabilities, instead of just likelihoods (which do not require the EM algorithm), is higher when the coverage and the minor allele frequency are lower; and 2) applications that require accurate genotypes should use coverages higher than 4, unless the minor allele frequency is always very low. The very high levels of genotype errors observed when the minor allele frequency is high and the coverage is low are an instrinsic problem of the limited amount of data available to infer the genotype. Even if the allele frequency and the allele sampling bias were known with accuracy, up to 50% of the genotypes predicted by maximum posterior probability are expected to be wrong when the coverage is 1 and the minor allele frequency is 0.5 (Figure S5 in Additional file 2). The fact that allele and genotype frequencies can be estimated accurately under rampant uncertainty of individual genotypes strongly encourages the use and further development of genotype-free methods, that take full advantage of low-coverage sequencing data.

Next, we checked *svgem* performance with different sample sizes from 10 to 1000 and a fixed SV frequency of 0.5, which is the one with higher sampling variance. Figure S1 in Additional file 2 shows that smaller samples, with a mean coverage of 4, also yield unbiased estimates with a precision comparable to the expected under accurate knowledge of genotypes.

**Figure 4:**
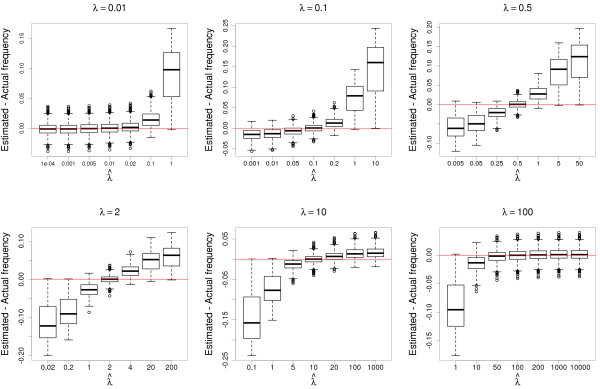
**Effect of the allele sampling bias on allele frequency estimates.** Accuracy and precision of the allele frequency estimates under biased sampling of alleles from heterozygous individuals, with accurate or inaccurate estimates of the allele sampling bias. The titles above the plots show the real values of *λ*. A red line is traced where the estimated and current allele frequencies are the same. Each combination of real and estimated *λ* values is simulated 1000 times, using random allele frequencies. All allele frequency estimates are run here with 1000 individuals sequenced at 4 × coverage, in order to reduce the dispersion of the estimates and make the bias caused by the misspecification of *λ* more visible.

Finally, we prove that arbitrarily high reference bias does not deviate the estimates from the true values, if *svgem* is informed of the bias. Figure 4 represents the accuracy and the biases of the estimates of the allele frequency for several combinations of the true (*λ*) and the estimated ($\hat{\lambda }$) values of the allele sampling bias. The estimates are always unbiased if $\hat{\lambda }=\lambda$, as expected. Interestingly, the estimates are also unbiased when *λ* is very low (≤0.01) or very high (≥100), and $\hat{\lambda }$ is even lower, or even higher, respectively. This implies that extreme values of *λ* can be effectively approximated by a wide range of values. The reason of this nice property is that at low coverage, the outcome of an extreme *λ* is always the same: none of a few observations gets to sample the disfavoured allele from a heterozyous sample. It is also worth noticing that rough approximations to *λ*, in the range between *λ* / 2 and 2*λ* produce only minor biases in allele estimates. Moreover, any estimated $\hat{\lambda }$ closer to the real value of *λ* than the default *λ* = 1 will improve the allele frequency estimates.

**Figure 5:**
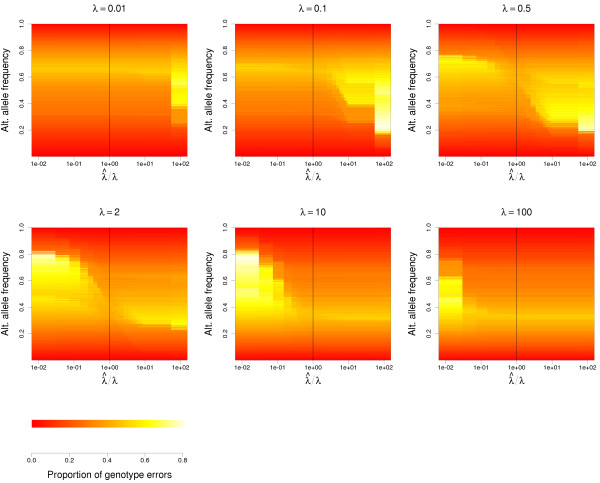
**Effect of the allele sampling bias on the accuracy of the most probable genotypes.** The heatmap represents the observed accuracy of the most probable genotypes in simulated datasets, as a function of the allele sampling bias (*λ*, above each plot), the ratio between the estimated and the true allele sampling bias ($\hat{\lambda }/\lambda$), and the frequency of the alternative allele. Each combination of real and estimated *λ* values is simulated 1000 times, using 500 individuals sequenced at 4 × coverage and a random allele frequency.

Figure 5 shows that the difficulty to predict individual genotypes varies in parallel with the difficulty in estimating the allele frequency in the presence of an uncertain allele sampling bias. While the accuracy of allele frequency estimates is mostly independent of the allele frequency (Figure 2), the accuracy in genotype prediction highly depends on the frequency of the genotypes, and therefore on the allele frequency. The heatmaps in Figure 5 represent the observed proportion of true genotypes that did not match the most probable genotype among 500 simulated diploid individuals, with a mean coverage of 4, as a function of the alternative allele frequency and the ratio between the estimated and the true allele sampling bias. The highest genotyping accuracy always happens when the true allele sampling bias is known ($\hat{\lambda }=\lambda$), and the most dramatic increase in genotyping errors happens when the estimated bias deviates in the direction opposite to the true bias.

It is also important to mention that the frequency of erroneous reference or alternative allele counts, *ε*_*r*_ and *ε*_*a*_, need to be either known or co-estimated from the data to get accurate estimates of allele or genotype frequencies. An erroneous count is a false observation of an allele, which should not contribute to the estimate of allele frequency. They are assumed to be less frequent than true counts. In practice, the accurate estimation of both the allele (or genotype) frequency and the frequency of erroneous counts is only feasible if there is enough information in the data. As a rule of thumb, when coverage is below 4, or the number of individuals is below 100, *a priori* estimates of *ε*_*r*_ and *ε*_*a*_ are highly recommended. They can be obtained from simulations, or estimated from a subset of individuals with high coverage, or empirically determined in a subset of homozygous individuals.

### Analysis of real data

**Figure 6:**
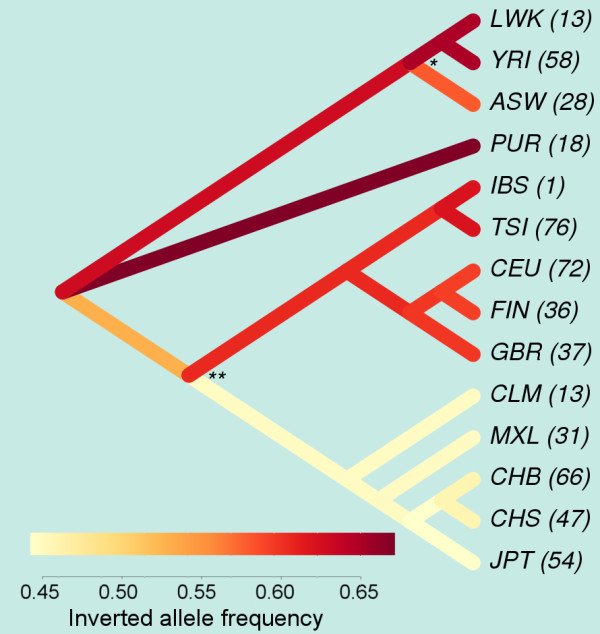
**Estimated global frequency of inversion HsInv0201 in human populations.** Allele frequencies of a polymorphic inversion on chromosome 5, calculated with *svgem*, are shown as colors on top of the topology of a Neighbor-joining tree of some human populations. The tree was built using the SNP-based, genome-wide, Hudson’s *F*_*ST*_s among the populations as distances [3]. In each bifurcation, the overall frequency was assiged to both sister clades if there were no significant differences. Significant changes are labeled with asterisks (*, *p*-value < 0.05; **, *p* - value < 0.001). The numbers of individuals analysed are shown in parentheses, and population labels are: LWK, Luhya (Kenia); YRI, Yoruba (Nigeria); ASW, African-American (Southwest, US); PUR, Puerto Rican; IBS, Iberian (Spain); TSI, Tuscan (Italy); CEU, Utah residents with Northern and Western European ancestry; FIN, Finnish; GBR, British (England, and Scotland); CLM, Colombian; MXL, Mexican; CHB, Han Chinese (Beijing, China); CHS, Southern Han Chinese; JPT, Japanese.

To test the performance of the algorithm on real data, we used as a model a previously unknown human inversion with simple breakpoints. Analysis of this inversion was part of a larger study to characterize and validate polymorphic inversions in human populations. In particular, the inversion selected (HsInv0201) is a 376 bp inversion in the Chr5q33.1 region, supported by paired-end mapping data of fosmids [39] or small DNA fragments [40,41]. By comparison of the HG18 Human Genome reference assembly [42] with the alternative human assemblies of Celera [43] and HuRef [44], it was found that the inverted allele includes two small deletions flanking the inversion and it was possible to locate the breakpoints (BP) to HG18 position chr5:147533233-147534432 (BP1) and chr5:147534809-147534971 (BP2), which correspond to the sequences deleted in the inverted chromosomes [45]. From there, we extracted 100 nucleotides-long *in silico* probes, in which the sequence change between the two orientations is located exactly in the middle (see Table S3 in Additional file 3). Then, we mapped the reads from 550 individuals from the 1000 Genomes Project [3] on these probes, using the program *BreakSeq*[38], and counted how many matched specifically the reference or the alternative breakpoints. In order to quantify the allele sampling bias, *λ*, we extracted all possibly informative reads from the 100 nucleotides probes (see Table S3 in Additional file 3), with the same length range as the real reads used (36–100 nucleotides), and used *BreakSeq* again to count how many of them mapped uniquely to either the reference or the alternative breakpoints. A negligible bias (*λ* = 1) was found. Erroneous counts were experimentally determined to be also negligible (see below), and their frequencies were set to 1.0×10^-5^, although several orders of magnitude of variation in this parameter did not alter the results significantly. From the allele counts and from these parameter values, *svgem* estimates a global alternative allele frequency of 0.55, and population-specific frequencies ranging from 0.45 to more than 0.65 (Figure 6). Among related individuals, only the oldest parents of a family were retained for the estimation of population parameters. Using a likelihood ratio test, we prove that Asian and native American populations have a significantly lower frequency of the alternative conformation than African and European populations (*p*-value =5.3 × 10^-5^).

To genotype experimentally the inversion, we used different pairs of primers specific for the reference orientation (A2-B2) or the inverted orientation (A4-C3 and B2-D1; Table S3 in Additional file 3) and carried out duplicate PCRs of each individual, both in simplex and multiplex format. In total, the 270 individuals of the Phase II of the HapMap Project were analyzed, including 90 Yoruba (YRI), 90 from European origin (CEU), 45 Chinese (CHB) and 45 Japanase (JPT), and the PCR results can be accessed in the InvFEST database [45]. Table S4 shows the observed genotypes and the genotype posterior probabilities calculated with *svgem* for the 122 individuals that were both genotyped by PCR and analysed with *BreakSeq* and *svgem*. The alternative allele frequencies determined experimentally or estimated with *svgem* in this subsample were, respectively, 0.545 (standard error 0.045), and 0.541. The most probable genotype determined by *svgem* matched the true genotype in 111 (91%) individuals, with only 1 error (out of 86) when the coverage is higher than 2. From the allele counts of homozygous individuals, it can be seen that the opposite allele is never observed, confirming that the rate of erroneous counts is negligible.

**Figure 7:**
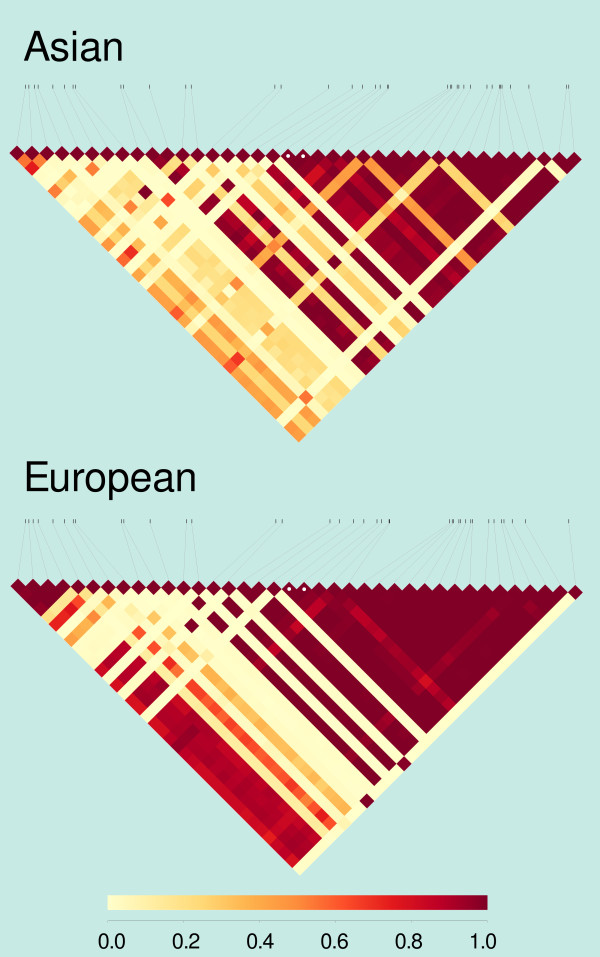
**Linkage disequilibrium patterns around inversion HsInv0201 in Asian and European populations.** The heatmap represents values of *r*^2^, a measure of linkage disequilibrium between pairs of SNPs or between SNPs and the breakpoints of inversion HsInv0201. The two breakpoints are marked with white spots. The SNP positions span chromosome 5 (hg19) bases 147549663 to 147556698 in Asians, and 147549507 to 147556942 in Europeans.

Once having accurate genotype likelihoods of this short inversion, it is possible to calculate its linkage disequilibrium with nearby SNPs, without having to know the true genotypes, neither of the inversion, nor of the SNPs. This allows the study of the association between structural variants and SNPs without having to rely on imputed genotypes, and without having to exclude SNPs with arbitrary coverage thresholds. The method to calculate the pairwise linkage disequilibrium statistic *r*^2^ from genotype likelihoods is implemented in *bcftools*[24], and requires an input VCF file with genotype likelihoods. We downloaded from the 1000 Genomes Project database a VCF file spanning 7 kb around the inversion, and manually combined it with the inversion itself, represented by two punctual variants at the positions of its breakpoints. Figure 7 shows how the two breakpoints are correctly determined to be in perfect linkage disequilibrium between them, and how the patterns of linkage among the inversion and the SNPs around it differ between European and Asian populations. Note that linkage disequilibrium estimates, let alone their comparison between populations, would be biased if imputed genotypes had been used, because imputation already assumes some linkage disequilibrium, not always measured in the population of interest.

## Conclusions

The development of methods to discover structural variants in individual genomes is giving way to population-level analyses. The most recently developed discovery tools, such as GASVPro [21] or CloudBreak [46], call the genotypes of the individuals analysed, instead of just reporting the variants discovered (a notable exception being ForestSV [20]). However, these individual-based methods require high coverage, and they are oblivious to the information present at the population level. While these methods are still useful in some applications, there is a current demand for efficient ways to analyse low-coverage population genomics data. Most studies still insist in genotyping the individuals, despite of the loss of data caused by arbitrary quality thresholds, and despite the circularity and biases associated with genotype imputation [1,47,48]. The alternative of using likelihood or Bayesian approaches, which take genotype uncertainty into account, is an optimal strategy to explore genetic diversity, since it does not require high coverage per individual, and allows the sequencing of more individuals at the same cost [49]. Not surprisingly, a new method to genotype indels from sequence data in polyploid genomes uses the same approach of likelihood calculation, frequency estimation through an expectation-maximization algorithm, and reporting of posterior probabilities [50]. However, this method does not consider any allele sampling bias, because it targets indels shorter than the reads used to distinguish the alleles. By including allele sampling bias in the genotype likelihood calculation, our program extends the applicability of these methods to the analysis of large structural variation. Furthermore, for the first time nucleotide and structural variation can be analysed in the same statistical framework, without having to rely on the accuracy of the genotypes.

Two of the key features of *svgem* are its simplicity and its few assumptions about the data, which make the program useful for a wide variety of data types. Any bi-allelic structural variant detected by sequenced paired-ends or split reads, including inversions, mobile element insertions, duplications, and deletions, can be analysed by *svgem*. Using simulations, we have shown that estimates of allele or genotype frequencies are accurate, even in the face of rampant allele sampling bias, that usually accompanies the detection of structural alleles. Finally, using data from the 1000 Genomes Project and PCR experiments, we prove its applicability to real data.

## Availability and requirements

**Project name:** svgem.

**Project home page:**http://grupsderecerca.uab.cat/cacereslab/content/resources.

**Operating system(s):** Platform independent.

**Programming language:** C++.

**Other requirements:** None.

**License:** GNU General Public License.

**Any restrictions to use by non-academics:** None.

## Abbreviations

EM: Expectation-maximization; kb: kilobase pairs; PC: Personal computer; HWE: Hardy-Weinberg equilibrium; PCR: Polymerase chain reaction; SNP: Single-nucleotide polymorphism; MAF: Minor allele frequency.

## Competing interests

The authors declare that they have no competing interests.

## Authors’ contributions

JILL wrote the program, run it on the simulated and real data, and coordinated the writing of the manuscript. DV retrieved the allele counts from the 1000 Genomes Project database. CA designed and oversaw the PCR experiments. MC designed the study and supervised all steps. All authors contributed to the writing. All authors read and approved the final manuscript.

## Supplementary Material

Additional file 1 **This is a plain text file containing the source code of *svgem*, in C++.** Click here for file

Additional file 2 **Additional text including *svgem*’s manual and some details on how the expectation-maximization algorithm is implemented.** Click here for file

Additional file 3 **Tables S3 and S4. Table S3.***In silico* probes and PCR primers. *In silico* probes including the two breakpoints, in both the reference and the inverted conformations, and primers used for PCR validations. **Table S4.** Experimental validation. Allele counts, genotype posterior probabilities obtained with *svgem*, and true genotypes determined by PCR, for inversion HsInv0201 in 122 individuals from the 1000 Genomes Project. Click here for file
