# Supplementary material for: Population genetic analysis of bi-allelic structural variants from low-coverage sequence data with an expectation-maximization algorithm
Source: BMC Bioinformatics. 2014 May 29;15:163. doi: 10.1186/1471-2105-15-163 (PMC4055234; doi:10.1186/1471-2105-15-163)
Supplement: Additional file 2 — Additional text including svgem ’s manual and some details on how the expectation-maximization algorithm is implemented. [file 1471-2105-15-163-S2.pdf]

Population genetic analysis of structural variants from low-coverage sequence data with an expectation-maximization algorithm. Manual and supplementary text.

José Ignacio Lucas-Lledó, David Vicente-Salvador,
Cristina Aguado, & Mario Cáceres

May 2, 2014

Installation

It has only been tested on Linux. Compile the source code typing the following:

```
g++ svgem.cpp -o svgem
```

Options

-i <string> Input file name (required). The input file must be a tab-delimited, text file with three or four columns. It contains the counts of reference and alternative alleles for all the individuals at one variable site. The columns are the following:

1. Sample, or individual identifier.
2. Reference count (float). It is the number of times the reference allele has been observed in this individual, in the locus being analysed. For example, for a structural variant detected with paired-end sequencing, it is the number of concordant pairs of reads compatible with the site being in standard conformation. If different qualities are assigned to the observations, the expected number of observations should be reported here, calculated as the summation of the probabilities of the counts being true.
3. Alternative count (float). It is the number of times the alternative allele has been observed in this individual and in the locus being analysed, or the expected number of true observations, if each observation has a different quality.

4. Ploidy of the locus in this individual (1 or 2). Optionally, the ploidy of the locus can be specified for each individual, so that variation in the X chromosome can be analysed in a mixture of males and females. If ploidy is specified in the input file, the option -p must be set to 0.

-o <string> Output file (optional; default, standard output).

-p <int> Ploidy (0, 1, or 2; default, 2). If this parameter is set to 1 or 2, all individuals are assumed to have the same ploidy, and the input file is assumed to have 3 columns. If this parameter is set to 0, the input file must be in the 4-columns format.

-b <float> Reference bias (default, 1.0). It is the ratio between the probability of observing the reference allele and the probability of observing the alternative allele from a heterozygous sample. The reference bias is not estimated by svgem, and needs to be provided here. The recommended way to estimate it is to perform simulations of the sequencing and mapping of reads from both the reference and the alternative alleles, and then use the ratio of reference to alternative counts observed.

-f <float> Initial value of the alternative allele frequency to start the iterations (default, 0.5). It is advised to run svgem more than once with different starting values, to make sure that the algorithm converges to the maximum-likelihood estimate. Setting -f implies the assumption of Hardy-Weinberg equilibrium.

-F <string> Comma separated initial values of genotype frequencies (default '0.25,0.5,0.25'). There must be no spaces between the values. They correspond to the genotypes: homozygous for the alternative (Alt/Alt), heterozygous (Ref/Alt), and homozygous for the reference (Ref/Ref). This is incompatible with -f, and allows to override the assumption of Hardy-Weinberg equilibrium. It is a good practice to re-run *svgem* with alternative initial values to ensure that the final estimates are the maximum-likelihood estimates.

-e <float> (Initial) estimate of the frequency of errors among counts of the alternative allele (default, 0.001). It is assumed to be low, and svgem stops and complains if it is equal or higher than 0.5. This limitation may prevent the estimation of very low alternative allele frequencies. This value is not optimized, unless the flag -s is set.

-E <float> (Initial) estimate of the frequency of errors among counts of the reference allele (default, 0.001). This is also assumed to be low, and not optimized unless -s is set.

-s Turn on the estimation of frequencies of errors (default, false). This flag determines the estimation of two more parameters, the initial values of which are set by -e and -E. This is not recommended on very low coverage data. Under some circumstances, it can prevent the convergence of the estimates.

-w Force Hardy-Weinberg equilibrium (default, false, unless using -f).

-m <int> Maximum number of iterations. The estimates keep being refined until the maximum number of iterations is reached, or the difference between consecutive estimates of the alternative allele frequency become smaller than the tolerance, whichever happens before.

-t <float> Tolerance, or maximum difference between consecutive estimates to declare convergence (default, 1.0e-06).

-l Report relative, $\log_{10}$ genotype likelihoods, instead of posterior probabilities, in the *svgem.log* output (see below).

Output

Two outputs are produced. The main output, sent to either the standard output or to the requested file (see Options), reports the refinement of the estimates along the iterations of the EM algorithm. It has 8 fields:

1. Iteration number.
2. Current estimate of the alternative allele frequency.
3. Current estimate of the frequency of homozygous alternative genotypes.
4. Current estimate of the frequency of heterozygous genotypes.
5. Current estimate of the frequency of homozygous reference genotypes.
6. Current estimate of the frequency of errors among alternative counts.
7. Current estimate of the frequency of errors among reference counts.
8. Log-likelihood of the current estimates.

The log-likelihood should always increase, and its final value can be used to perform likelihood ratio tests (see below).

The second output is an extended version of the input file with three additional fields, reporting the posterior probabilities of the three genotypes in each individual (Alt/Alt, Alt/Ref, and Ref/Ref), based on the last estimates of the allele frequency and the error rates. It is always named *svgem.log*. For

Table 1: Notation.

k	Total number of allele observations, or counts, in one individual.
l	Number of times the reference allele is observed in one individual ($l \leq k$).
m	Ploidy.
g	Number of reference alleles in the genotype ($g \leq m$).
λ	Allele sampling bias in heterozygous individuals.
ϵ_r	Frequency of erroneous counts among reference counts.
ϵ_a	Frequency of erroneous counts among alternative counts.

Table 2: Likelihoods of the three diploid genotypes ($m = 2$).

g	$\mathcal{L}(g)$
0	$\epsilon_r^l(1-\epsilon_a)^{k-l}$
1	$\left(\frac{1}{1+\lambda}\right)^k (\epsilon_r + \lambda - \lambda\epsilon_r)^l(1-\epsilon_a+\lambda\epsilon_a)^{k-l}$
2	$(1-\epsilon_r)^l\epsilon_a^{k-l}$

hemizygous samples, the first and the third of these additional fields are used to represent the probabilities of genotypes Alt/0 and Ref/0, respectively. The genotype likelihoods, instead of the posterior probabilities, can be requested in this output (see option -l).

Derivation of the likelihood functions

Following the notation in [Li, 2011] (see Table 1), we refer to a genotype by its number of reference alleles, $g \in \{0, 1 \dots m\}$, where m is the ploidy, usually 2. We assume that variants are biallelic, so that $m - g$ is the number of alternative alleles in the genotype. Table 2 shows the likelihoods of the three diploid genotypes.

The likelihood functions shown on table 2 are derived as follows. k is the total number of observations of the two possible (structural) alleles. That is, the coverage of the structural variant. Each observation is a direct evidence of one or the other allele, whether these are single or paired-end reads mapping on the breakpoints or on the inserted or deleted region. Allele observations are independent among them, and they may have different qualities (that is, different probabilities of being erroneous). The likelihood of a genotype is the probability of observing the reference allele exactly l times ($l \leq k$) and

the alternative allele, $k-l$ times, with the corresponding probabilities of the observations being erroneous, given that the genotype is g. An erroneous observation would be the observation of the allele that was not actually sequenced, but the other, for example, due to mapping errors.

Given the assumed independence among observations, the likelihood is the product of the probabilities of all individual observations. The probability of observing an allele x with a given quality is equal to: $(1-P(x))\epsilon_j + P(x)(1-\epsilon_j)$, where $P(x)$ is the probability of sampling that allele during sequencing, and ϵ_j is the probability of the observation being erroneous, according to the its quality.

The probability of sampling one of the two alleles during sequencing is usually assumed to be proportional to the number of chromosomes carrying that allele in the genotype: g for the reference allele, and $m-g$ for the alternative. In the case of a sampling bias of λ, any chromosome with the reference allele is λ times as likely to be sequenced as a chromosome with the alternative allele. Thus, the probability of sampling the reference allele is $\frac{\lambda g}{m+\lambda g-g}$. For example, in a triploid individual ($m=3$), with two homologous chromosomes carrying the reference allele of a deletion, and the third chromosome carrying the alternative (deleted) allele ($g=2$), the probability of sampling the reference allele could be magnified from the naive $\frac{2}{3}$ to the actual $\frac{4}{5}$, if $\lambda=2$, because each reference allele counts twice as much as an alternative. Putting it all together, and letting the first l observations be of the reference allele, we have:

$$\mathcal{L}(g) = \prod_{j=1}^{l}\left[\frac{m-g}{m+\lambda g-g}\epsilon_j + \frac{\lambda g}{m+\lambda g-g}(1-\epsilon_j)\right]\prod_{j=l+1}^{k}\left[\frac{m-g}{m+\lambda g-g}(1-\epsilon_j) + \frac{\lambda g}{m+\lambda g-g}\epsilon_j\right]$$

$$\mathcal{L}(g) = \left(\frac{1}{m+\lambda g-g}\right)^k \prod_{j=1}^{l}\left[(m-g)\epsilon_j + \lambda g(1-\epsilon_j)\right]\prod_{j=l+1}^{k}\left[(m-g)(1-\epsilon_j) + \lambda g\epsilon_j\right]$$

The equation above becomes identical to Li's equation 2 if $\lambda=1$. In order to obtain the likelihood functions shown on table 2, we first treat all the observations of the same allele as having the same, average, probability of being errnoeous: ϵ_r in the case of a reference observation, and ϵ_a in the case of an alternative observation. And then, we fix $m=2$ for diploid variants. Because $\mathcal{L}(0)$ and $\mathcal{L}(m)$ are independent of m, the two possible genotypes in a hemizygous (sex) chromosome ($g=0$, and $g=1$) have the same likelihood functions as the genotypes $g=0$, and $g=2$ of diploid variants.

Implementation of the expectation-maximization algorithm

Treating the genotypes as missing values, we implement an expectation-maximization (EM) method to estimate either the alternative allele fre-

quency, ψ, under the assumption of Hardy-Weinberg equilibrium, or the genotype frequencies ψ_g (with $g \in \{0, 1, 2\}$ for diploids) or ϕ_g (with $g \in \{0, 1\}$, for hemizygous individuals), and eventually the proportions of errors among reference (ϵ_r) and alternative (ϵ_a) counts. Note that ψ_g is the frequency of genotype g *among diploids*, and ϕ_g is the frequency of genotype g *among hemizygous individuals*. The EM algorithm is an iterative estimation of the parameters that gets closer to the maximum likelihood estimates in every iteration. Let Θ represent the parameters being estimated. Several good manuals show that the next $(t+1)$ estimate of the parameters is obtained from the current (t) estimates by the following expression:

$$\begin{aligned}\Theta^{(t+1)} &= \arg\max_{\Theta} \left\{ \mathrm{E}_{\mathbf{G}|\mathbf{X},\Theta^{(t)}} \{\log P(\mathbf{X}, \mathbf{G} \mid \Theta)\} \right\} \\ &= \arg\max_{\Theta} \left\{ \sum_{\mathbf{G}} P(\mathbf{G} \mid \mathbf{X}, \Theta^{(t)}) \cdot \log P(\mathbf{X}, \mathbf{G} \mid \Theta) \right\}\end{aligned}$$

where, $\mathbf{X}$ is the whole set of allele counts across individuals, and the summation is over all possible combinations of genotypes among individuals ($\mathbf{G}$). This becomes tractable when, following Gupta and Chen [2010], the summation is performed over individuals and genotypes, because the data $\mathbf{X}$ is composed of N independent and identically distributed samples, namely the individuals:

$$\Theta^{(t+1)} = \arg\max_{\Theta} \left\{ \sum_{i=1}^{N} \sum_{g=0}^{m_i} P(g \mid \mathbf{X}_i, \Theta^{(t)}) \cdot \log P(\mathbf{X}_i, g \mid \Theta) \right\} \qquad (1)$$

$$\Theta^{(t+1)} = \arg\max_{\Theta} \left\{ \sum_{i=1}^{N} \sum_{g=0}^{m_i} P(g \mid \mathbf{X}_i, \Theta^{(t)}) \cdot \log(P(\mathbf{X}_i \mid g, \Theta) \cdot P(g \mid \Theta)) \right\}$$

Using Bayes theorem and the likelihood function described in table 2, the function in curly brackets can be expressed in terms of the parameters ϵ_r, ϵ_a, and either ψ or ψ_g, depending on whether Hardy-Weinberg equilibrium is assumed or not. If not, $P(g|\Theta) = \psi_g$ (if diploid; ϕ_g, otherwise), and the conditions $\sum_g \psi_g = 1$ and $\sum_g \phi_g = 1$ must be used during optimization, by means of Lagrangian multipliers. Then, the next values of the parameters

can be found to be the following:

$$\psi^{(t+1)} = \frac{2D_0^{(t)} + D_1^{(t)} + H_0^{(t)}}{2(D_2^{(t)} + D_1^{(t)} + D_0^{(t)}) + H_0^{(t)} + H_1^{(t)}}$$

$$\psi_g^{(t+1)} = \frac{D_g^{(t)}}{D}$$

$$\phi_g^{(t+1)} = \frac{H_g^{(t)}}{H}$$

$$\epsilon_a^{(t+1)} = \frac{A_2^{(t)}}{A_0^{(t)} + A_2^{(t)}}, \qquad \text{if} \lambda = 1$$

$$\epsilon_r^{(t+1)} = \frac{R_0^{(t)}}{R_0^{(t)} + R_2^{(t)}}, \qquad \text{if} \lambda = 1$$

In the equations above, $D_g^{(t)}$ is the tth estimate of the total number of diploid individuals with genotype g, and $H_g^{(t)}$ is the tth estimate of the total number of hemizygous individuals with genotype g. That is, they are the summations of the posterior probabilities of genotype g over the respective kind of individuals. D and H are the total number of diploid and hemizygous individuals, respectively, where $D + H = N$. $A_g^{(t)}$ is the tth estimate of the total number of alternative counts coming from hemizygous and homozygous individuals for either the alternative ($g = 0$) or the reference ($g = 2$) allele. Finally, $R_g^{(t)}$ is the tth estimate of the total number of reference counts that come from hemizygous and homozygous individuals for either the alternative

($g = 0$) or the reference ($g = 2$) allele. Explicitly,

$$D_g^{(t)} = \sum_{i:m_i=2}^{N} P(g \mid \mathbf{X_i}, \Theta^{(t)})$$

$$H_g^{(t)} = \sum_{i:m_i=1}^{N} P(g \mid \mathbf{X_i}, \Theta^{(t)})$$

$$A_0^{(t)} = \sum_{i}^{N} P(g = 0 \mid \mathbf{X_i}, \Theta^{(t)}) \cdot (k_i - l_i)$$

$$A_1^{(t)} = \sum_{i:m_i=2}^{N} P(g = 1 \mid \mathbf{X_i}, \Theta^{(t)}) \cdot (k_i - l_i)$$

$$A_2^{(t)} = \sum_{i:m_i=1}^{N} P(g = 1 \mid \mathbf{X_i}, \Theta^{(t)}) \cdot (k_i - l_i) + \sum_{i:m_i=2}^{N} P(g = 2 \mid \mathbf{X_i}, \Theta^{(t)}) \cdot (k_i - l_i)$$

$$R_0^{(t)} = \sum_{i}^{N} P(g = 0 \mid \mathbf{X_i}, \Theta^{(t)}) \cdot l_i$$

$$R_1^{(t)} = \sum_{i:m_i=2}^{N} P(g = 1 \mid \mathbf{X_i}, \Theta^{(t)}) \cdot l_i$$

$$R_2^{(t)} = \sum_{i:m_i=1}^{N} P(g = 1 \mid \mathbf{X_i}, \Theta^{(t)}) \cdot l_i + \sum_{i:m_i=2}^{N} P(g = 2 \mid \mathbf{X_i}, \Theta^{(t)}) \cdot l_i$$

When there is sampling bias in heterozygous individuals, $\lambda \neq 1$, and the next values of the proportions of errors among reference (ϵ_r) and alternative (ϵ_a) counts are the results of the two quadratic equations below:

$$\begin{aligned}
(R_0^{(t)}\lambda + R_1^{(t)}\lambda + R_2^{(t)}\lambda - R_0^{(t)} - R_1^{(t)} - R_2^{(t)})\epsilon_r^2 + \\
+(R_0^{(t)} + R_1^{(t)} - 2R_0^{(t)}\lambda - R_1^{(t)}\lambda - R_2^{(t)}\lambda)\epsilon_r + \\
+R_0^{(t)}\lambda = 0 \\
(A_0^{(t)} + A_1^{(t)} + A_2^{(t)} - A_0^{(t)}\lambda - A_1^{(t)}\lambda - A_2^{(t)}\lambda)\epsilon_a^2 + \\
+(A_1^{(t)}\lambda + A_2^{(t)}\lambda - A_0^{(t)} - A_1^{(t)} - 2A_2^{(t)})\epsilon_a + \\
A_2^{(t)} = 0
\end{aligned}$$

Among the possible solutions, $\epsilon_r = \epsilon_a = 0$, $\epsilon_r = \epsilon_a = 1$, $\epsilon_r = 1/(1 + \lambda)$, and $\epsilon_a = 1/(1 - \lambda)$ are excluded. In practice, it is assumed that the erroneous counts are a minority, and the program halts when $\epsilon_r \geq 0.5$ or $\epsilon_a \geq 0.5$. This can prevent the correct estimation of very low allele frequencies, in the presence of erroneous counts, as should be expected.

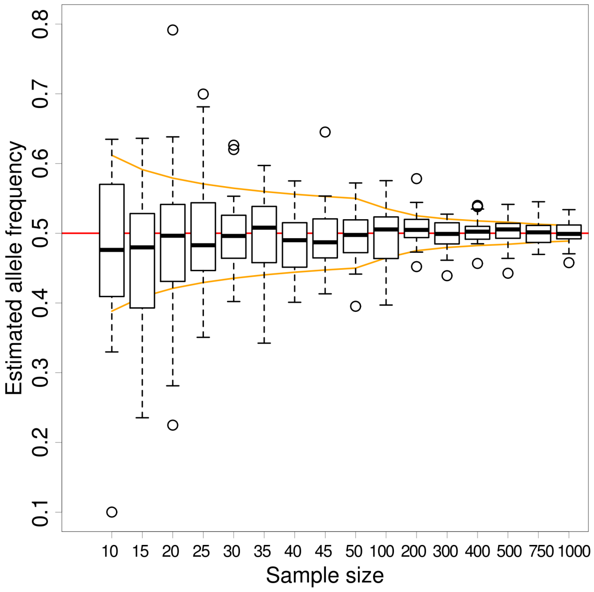

Figure S1: Precision of the estimates of allele frequency from samples of different sizes, compared to the expected precision (orange lines) with known genotypes. Again, for each sample size, we performed 50 simulations. Here, the mean coverage is 4.

Effect of sample size on allele frequency estimates

Figure S1 shows that sample size affects the precision of the allele frequency estimates as expected, without imparing the accuracy: while smaller samples produce necessarily less precise estimates, they are not biased.

Accuracy of genotype frequencies

svgem can be run with or without the assumption of Hardy-Weinberg equilibrium. When the equilibrium is assumed, genotype frequencies follow from the alternative allele frequency, which is the only parameter being estimated, in this case. When the equilibrium is not assumed, however, the frequencies of the three genotypes among diploid individuals (and the frequencies of the two possible genotypes among hemizygous individuals, if any) are estimated. The estimation of genotype frequencies without assuming Hardy-Weinberg equilibrium is necessary for some applications, such as estimating heterozygosity and inbreeding coefficients. The fact of estimating more parameters with the same amount of data entails that the precision cannot be as high as when estimating only one parameter. In comparison with Figure 2 of the main text, Figures S2–S4 show that genotype frequencies are more challeng-

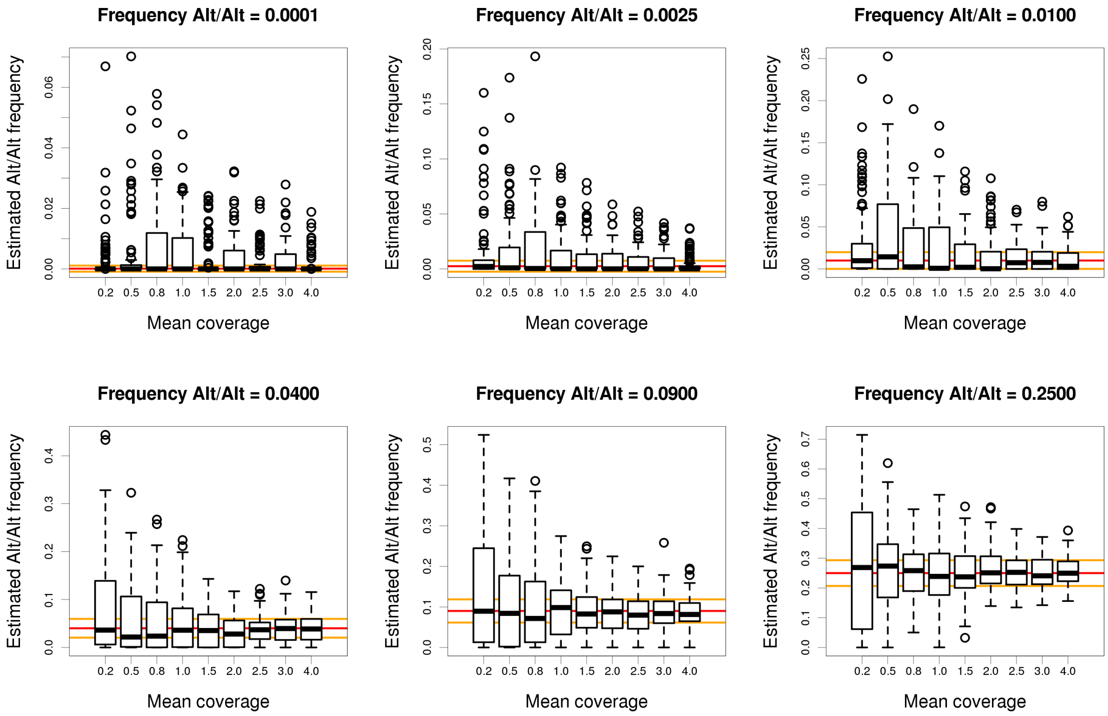

Figure S2: Estimates of the frequency of the genotype homozygous for the alternative allele (Alt/Alt) obtained by *svgem* from random samples of 100 diploid individuals, from populations with Alt/Alt frequencies between 0.0001 and 0.25 (upper label of each plot), for mean coverage depths between 0.2 and 4. Each combination of Alt/Alt frequency and depth of coverage was simulated 100 times. The red line indicates the true genotype frequency, and the orange lines indicate the expected standard error of the true frequency, from a sample of 200 chromosomes.

ing to estimate.

Accuracy of the predicted genotypes

For some analyses, the genotypes need to be known, because genotype-free methods have not been developed yet. For example, coalescence-based studies may always require accurate genotypes, because they already deal with enough uncertainty about the genealogy. In these situations, higher levels of coverage are necessary to achieve genotype accuracy. With high coverage, the benefits of running the expectation-maximization algorithm implemented in *svgem*, as oposed to simply using the most likely genotype of each individual, diminish. Indeed, *svgem* was designed to take advantage of low-coverage

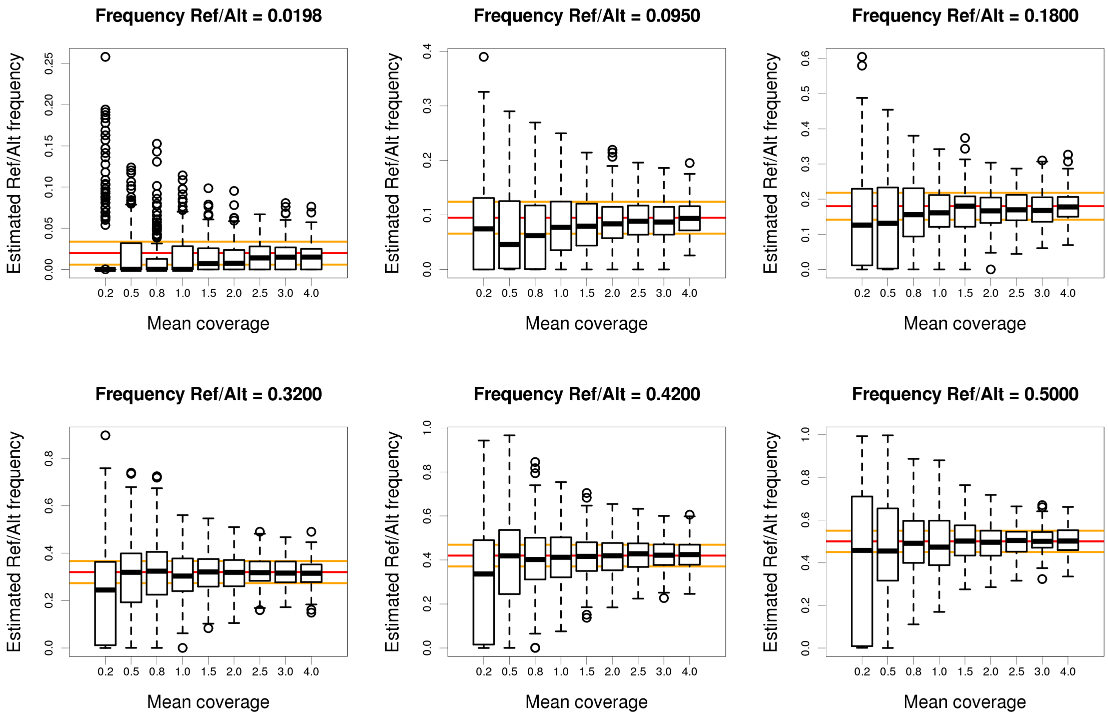

Figure S3: Estimates of the frequency of the heterozygous genotype (Ref/Alt) obtained by *svgem* from the same random samples of 100 diploid individuals used in Figure S2, where Ref/Alt frequencies range between 0.0198 and 0.5 (upper label of each plot). The red line indicates the true genotype frequency, and the orange lines indicate the expected standard error of the true frequency, from a sample of 200 chromosomes.

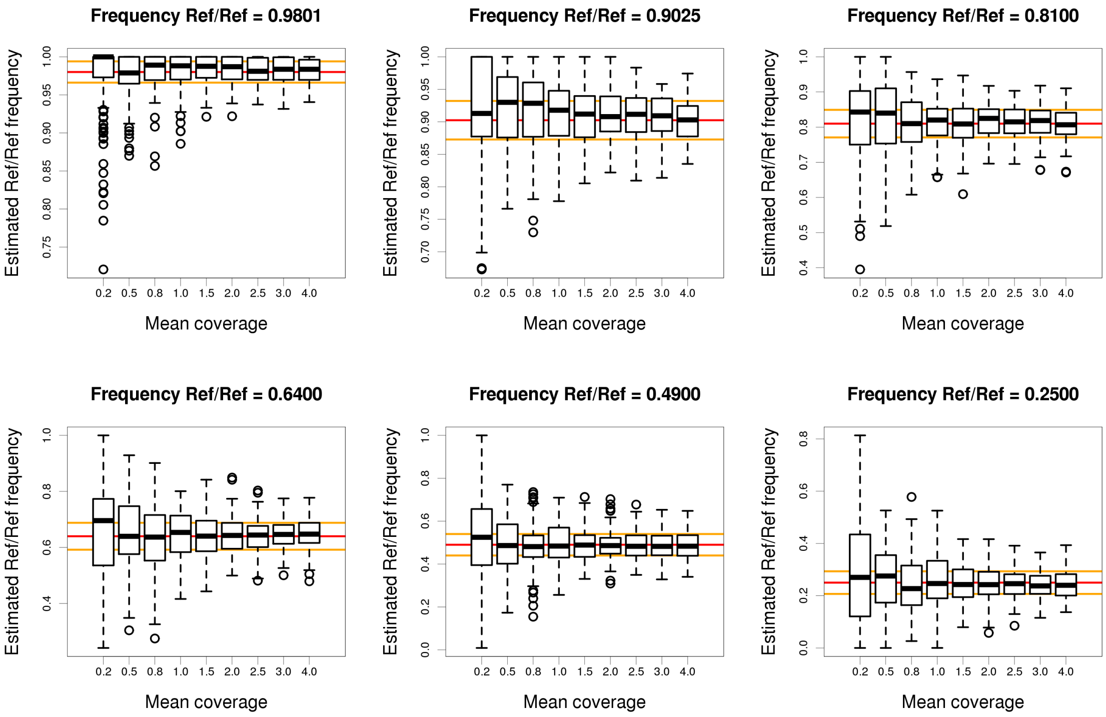

Figure S4: Estimates of the frequency of the genotype homozygous for the reference allele (Ref/Ref) obtained by *svgem* from the same samples of 100 diploid individuals used in Figures S2 and S3, where Ref/Ref frequencies range between 0.25 and 0.9801 (upper label of each plot). The red line indicates the true genotype frequency, and the orange lines indicate the expected standard error of the true frequency, from a sample of 200 chromosomes.

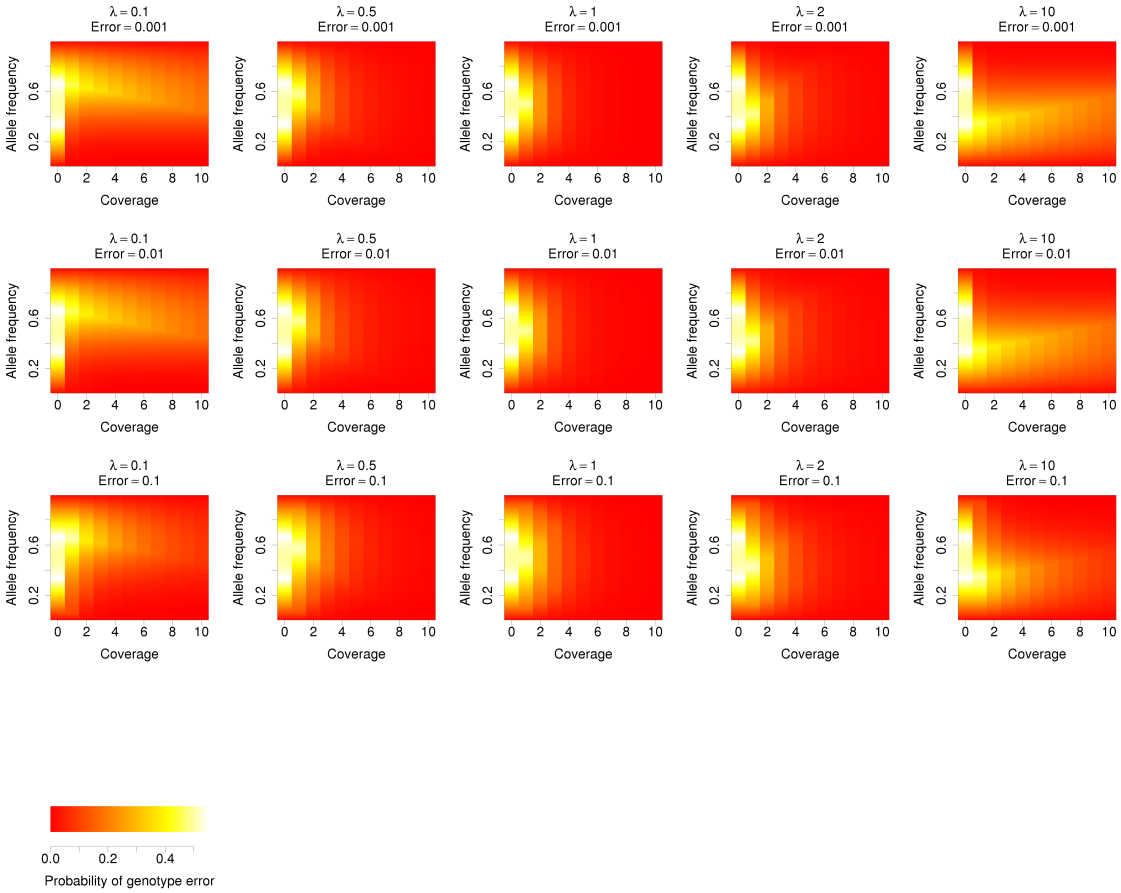

Figure S5: Expected probability of the true diploid genotype being different from the most probable genotype after sampling its alleles between 0 and 10 times (Coverage), with a given allele bias (λ) and with a finite probability of spurious erroneous observations (Error).

datasets, where genotypes are difficult or impossible to guess. The performance of *svgem* at predicting individual genotypes (Figures 3 and 5 of the main text) should be compared with the theoretical expectation. Given a number of allele observations in a diploid individual (coverage), the probability of a wrong genotype having higher posterior probability than the true one depends on the allele frequency, the rates of erroneous observations, and the allele sampling bias (λ). Figure S5 shows the numerical solutions of this function, which represent the expected proportions of individuals with a most probable genotype different from their true genotype, when allele frequency and allele sampling bias are known with accuracy. Interestingly, extreme allele sampling biases impair genotype prediction even with moderate depth of coverage.

References

G. R. Abecasis, A. Auton, L. D. Brooks, M. A. DePristo, R. M. Durbin, R. E. Handsaker, H. M. Kang, G. T. Marth, and G. A. McVean. An integrated map of genetic variation from 1,092 human genomes. *Nature*, 491(7422):56–65, Nov. 2012.

S.-M. Ahn, T.-H. Kim, S. Lee, D. Kim, H. Ghang, et al. (21 co-authors). The first korean genome sequence and analysis: full genome sequencing for a socio-ethnic group. *Genome Res.*, 19(9):1622–1629, Sept. 2009.

M. R. Gupta and Y. Chen. Theory and use of the EM algorithm. *Foundations and Trends in Signal Processing*, 4(3):223–296, 2010.

J. M. Kidd, G. M. Cooper, W. F. Donahue, H. S. Hayden, N. Sampas, et al. (46 co-authors). Mapping and sequencing of structural variation from eight human genomes. *Nature*, 453(7191):56–64, May 2008.

H. Y. K. Lam, X. J. Mu, A. M. Stütz, A. Tanzer, P. D. Cayting, M. Snyder, P. M. Kim, J. O. Korbel, and M. B. Gerstein. Nucleotide-resolution analysis of structural variants using BreakSeq and a breakpoint library. *Nat Biotech*, 28(1):47–55, Jan. 2010.

S. Levy, G. Sutton, P. C. Ng, L. Feuk, A. L. Halpern, et al. (31 co-authors). The diploid genome sequence of an individual human. *PLoS Biol*, 5(10): e254, Sept. 2007.

H. Li. A statistical framework for SNP calling, mutation discovery, association mapping and population genetical parameter estimation from sequencing data. *Bioinformatics*, 27(21):2987–2993, 2011.

K. J. McKernan, H. E. Peckham, G. L. Costa, S. F. McLaughlin, Y. Fu, et al. (45 co-authors). Sequence and structural variation in a human genome uncovered by short-read, massively parallel ligation sequencing using two-base encoding. *Genome Res.*, 19(9):1527–1541, Sept. 2009.

J. C. Venter, M. D. Adams, E. W. Myers, P. W. Li, R. J. Mural, et al. (247 co-authors). The sequence of the human genome. *Science*, 291(5507): 1304–1351, Feb. 2001.

Z. Yang. *Computational molecular evolution.* Oxford University Press, 2006.
