# Supplementary material for: Population genetic analysis of bi-allelic structural variants from low-coverage sequence data with an expectation-maximization algorithm
Source: BMC Bioinformatics. 2014 May 29;15:163. doi: 10.1186/1471-2105-15-163 (PMC4055234; doi:10.1186/1471-2105-15-163)
Supplement: Additional file 3 — Tables S3 and S4. Table S3.In silico probes and PCR primers. In silico probes including the two breakpoints, in both the reference and the inverted conformations, and primers used for PCR validations. Table S4. Experimental validation. Allele counts, genotype posterior probabilities obtained with svgem, and true genotypes determined by PCR, for inversion HsInv0201 in 122 individuals from the 1000 Genomes Project. [file 1471-2105-15-163-S3.pdf]

Population genetic analysis of structural variants from low-coverage sequence data with an expectation-maximization algorithm. Tables S3, and S4.

José Ignacio Lucas-Lledó, David Vicente-Salvador,
Cristina Aguado, & Mario Cáceres

October 24, 2013

Table S3: *In silico* probes and primers.

Name	Sequence (5'-3')
In silico probes	
Reference BP1	ATTGACTTCCACTCGAGAAGCTCTCAAATATCCCTT CAGGCCCTGAATTATTTTTTATTTTAAGAAAAATTG TAGTTTTGCTTTCTCTTAAATATCTTCA
Reference BP2	CAGGTCATCCCCACTAATAAGTAATTCAGCCAAAGT GTGAGAGTATTAACAGAATAGTTGAGGATTGACATG CCTCACACATCACAAACTCGGACCTGAT
Inverted BP1	CAGGGCATTAGTCTTAGTGATCTTGCACTTTACAAC TTTGAAATAGGAAAGTTAATTCCCTCACACTTTGGC TGAATTACTTATTAGTGGGGATGACCTG
Inverted BP2	GAAGATATTTAAGAGAAAGCAAAACTACAATTTTTC TTAAAATAAAAAAATAGTCAATTCCCTTTACACTCT GGATCTTAGTTTTTCCAAATATAAAACC
PCR primers	
HsInv201-A2	ATCAACCTTGAAGCCAGGAA
HsInv201-A4	CCCCTTCTTTCCAAATTCCT
HsInv201-B2	CTCCAATCTGATGCCATGG
HsInv201-C3	GCTCAGGTCATCCCCACTAA
HsInv201-D1	TAAAAAGCGCAAGAGGGAGA

Table S4: Counts of reference and alternative alleles, posterior probabilities of genotypes and true genotypes determined by PCR, for inversion HsInv0201 in 122 human individuals.

	**Allele counts**		**Posterior probabilities**			**True**
**Individual**	**Ref**	**Alt**	**Alt/Alt**	**Ref/Alt**	**Ref/Ref**	**genotypes**
NA06994	1	0	0.0001	0.5620	0.4380	Ref/Alt
NA07048	4	2	0.0000	1.0000	0.0000	Ref/Alt
NA07056	1	2	0.0004	0.9996	0.0000	Ref/Alt
NA07357	1	1	0.0002	0.9996	0.0002	Ref/Alt
NA10847	2	3	0.0000	1.0000	0.0000	Ref/Alt
NA10851	2	3	0.0000	1.0000	0.0000	Ref/Alt
NA11829	4	4	0.0000	1.0000	0.0000	Ref/Alt
NA11830	3	0	0.0000	0.2429	0.7571	Ref/Ref
NA11831	4	1	0.0000	0.9988	0.0012	Ref/Alt
NA11992	0	7	0.9859	0.0141	0.0000	Alt/Alt
NA11993	1	2	0.0004	0.9996	0.0000	Ref/Alt
NA11994	3	6	0.0000	1.0000	0.0000	Ref/Alt
NA11995	0	5	0.9459	0.0541	0.0000	Alt/Alt
NA12003	1	5	0.0035	0.9965	0.0000	Ref/Alt
NA12004	1	0	0.0001	0.5620	0.4380	Ref/Ref
NA12043	0	4	0.8974	0.1026	0.0000	Alt/Alt
NA12044	6	0	0.0000	0.0386	0.9614	Ref/Ref
NA12144	2	0	0.0000	0.3908	0.6092	Ref/Ref
NA12154	0	4	0.8974	0.1026	0.0000	Alt/Alt
NA12155	9	1	0.0000	0.9617	0.0383	Ref/Alt
NA12249	1	2	0.0004	0.9996	0.0000	Ref/Alt
NA12716	9	0	0.0000	0.0050	0.9950	Ref/Ref
NA12717	0	11	0.9991	0.0009	0.0000	Alt/Alt
NA12750	2	4	0.0000	1.0000	0.0000	Ref/Alt
NA12751	0	7	0.9859	0.0141	0.0000	Alt/Alt
NA12761	5	2	0.0000	1.0000	0.0000	Ref/Alt
NA12763	0	4	0.8974	0.1026	0.0000	Alt/Alt
NA18501	0	10	0.9982	0.0018	0.0000	Alt/Alt
NA18502	1	3	0.0009	0.9991	0.0000	Ref/Alt
NA18504	2	0	0.0000	0.3908	0.6092	Ref/Alt
NA18505	2	6	0.0000	1.0000	0.0000	Ref/Alt
NA18508	0	8	0.9929	0.0071	0.0000	Alt/Alt
NA18516	0	3	0.8139	0.1861	0.0000	Alt/Alt
NA18517	2	0	0.0000	0.3908	0.6092	Ref/Ref
NA18522	0	2	0.6862	0.3138	0.0000	Alt/Alt
NA18523	2	4	0.0000	1.0000	0.0000	Ref/Alt
NA18526	1	3	0.0009	0.9991	0.0000	Ref/Alt

Continued on next page

Table S4 – continued from previous page

Individual	Allele counts Ref	Alt	Posterior probabilities Alt/Alt	Ref/Alt	Ref/Ref	True genotypes
NA18532	1	4	0.0017	0.9983	0.0000	Ref/Alt
NA18537	0	3	0.8139	0.1861	0.0000	Alt/Alt
NA18542	0	2	0.6862	0.3138	0.0000	Ref/Alt
NA18547	0	4	0.8974	0.1026	0.0000	Alt/Alt
NA18550	0	2	0.6862	0.3138	0.0000	Ref/Alt
NA18552	0	6	0.9722	0.0278	0.0000	Alt/Alt
NA18555	2	2	0.0000	1.0000	0.0000	Ref/Alt
NA18558	5	0	0.0000	0.0743	0.9257	Ref/Alt
NA18561	0	6	0.9722	0.0278	0.0000	Alt/Alt
NA18562	1	3	0.0009	0.9991	0.0000	Ref/Alt
NA18563	1	2	0.0004	0.9996	0.0000	Ref/Alt
NA18564	2	1	0.0000	0.9997	0.0003	Ref/Alt
NA18566	6	0	0.0000	0.0386	0.9614	Ref/Ref
NA18570	0	3	0.8139	0.1861	0.0000	Alt/Alt
NA18571	5	0	0.0000	0.0743	0.9257	Ref/Ref
NA18573	2	0	0.0000	0.3908	0.6092	Ref/Ref
NA18576	3	0	0.0000	0.2429	0.7571	Ref/Ref
NA18577	2	3	0.0000	1.0000	0.0000	Ref/Alt
NA18579	7	0	0.0000	0.0197	0.9803	Ref/Ref
NA18582	2	0	0.0000	0.3908	0.6092	Ref/Ref
NA18592	3	1	0.0000	0.9994	0.0006	Ref/Alt
NA18603	1	0	0.0001	0.5620	0.4380	Ref/Alt
NA18605	1	0	0.0001	0.5620	0.4380	Ref/Alt
NA18608	3	3	0.0000	1.0000	0.0000	Ref/Alt
NA18609	0	1	0.5223	0.4777	0.0000	Ref/Alt
NA18611	2	0	0.0000	0.3908	0.6092	Ref/Alt
NA18612	0	5	0.9459	0.0541	0.0000	Alt/Alt
NA18620	1	1	0.0002	0.9996	0.0002	Ref/Alt
NA18622	1	2	0.0004	0.9996	0.0000	Ref/Alt
NA18623	0	2	0.6862	0.3138	0.0000	Ref/Alt
NA18624	0	2	0.6862	0.3138	0.0000	Ref/Alt
NA18632	8	0	0.0000	0.0099	0.9901	Ref/Ref
NA18633	1	2	0.0004	0.9996	0.0000	Ref/Alt
NA18635	0	5	0.9459	0.0541	0.0000	Alt/Alt
NA18636	5	0	0.0000	0.0743	0.9257	Ref/Ref
NA18637	2	2	0.0000	1.0000	0.0000	Ref/Alt
NA18856	5	0	0.0000	0.0743	0.9257	Ref/Ref
NA18858	2	0	0.0000	0.3908	0.6092	Ref/Ref
NA18861	0	2	0.6862	0.3138	0.0000	Alt/Alt
NA18870	3	4	0.0000	1.0000	0.0000	Ref/Alt

Continued on next page

Table S4 – continued from previous page

	Allele counts		Posterior probabilities			True
Individual	Ref	Alt	Alt/Alt	Ref/Alt	Ref/Ref	genotypes
NA18871	1	2	0.0004	0.9996	0.0000	Ref/Alt
NA18912	0	1	0.5223	0.4777	0.0000	Alt/Alt
NA18940	4	5	0.0000	1.0000	0.0000	Ref/Alt
NA18943	1	8	0.0272	0.9728	0.0000	Ref/Alt
NA18944	1	4	0.0017	0.9983	0.0000	Ref/Alt
NA18945	2	0	0.0000	0.3908	0.6092	Ref/Ref
NA18947	6	0	0.0000	0.0386	0.9614	Ref/Ref
NA18948	9	0	0.0000	0.0050	0.9950	Ref/Ref
NA18949	0	4	0.8974	0.1026	0.0000	Alt/Alt
NA18951	3	2	0.0000	1.0000	0.0000	Ref/Alt
NA18952	7	0	0.0000	0.0197	0.9803	Ref/Ref
NA18953	10	0	0.0000	0.0025	0.9975	Ref/Ref
NA18956	3	5	0.0000	1.0000	0.0000	Ref/Alt
NA18959	4	3	0.0000	1.0000	0.0000	Ref/Alt
NA18960	0	5	0.9459	0.0541	0.0000	Alt/Alt
NA18961	1	0	0.0001	0.5620	0.4380	Ref/Alt
NA18965	1	2	0.0004	0.9996	0.0000	Ref/Alt
NA18971	1	0	0.0001	0.5620	0.4380	Ref/Ref
NA18973	2	1	0.0000	0.9997	0.0003	Ref/Alt
NA18975	1	0	0.0001	0.5620	0.4380	Ref/Alt
NA18980	0	1	0.5223	0.4777	0.0000	Alt/Alt
NA18981	1	1	0.0002	0.9996	0.0002	Ref/Alt
NA18990	5	4	0.0000	1.0000	0.0000	Ref/Alt
NA18998	1	0	0.0001	0.5620	0.4380	Ref/Ref
NA18999	2	3	0.0000	1.0000	0.0000	Ref/Alt
NA19000	0	6	0.9722	0.0278	0.0000	Alt/Alt
NA19003	0	6	0.9722	0.0278	0.0000	Alt/Alt
NA19005	3	5	0.0000	1.0000	0.0000	Ref/Alt
NA19007	7	4	0.0000	1.0000	0.0000	Ref/Alt
NA19012	2	0	0.0000	0.3908	0.6092	Ref/Ref
NA19093	0	2	0.6862	0.3138	0.0000	Alt/Alt
NA19098	0	8	0.9929	0.0071	0.0000	Alt/Alt
NA19099	0	7	0.9859	0.0141	0.0000	Alt/Alt
NA19102	0	5	0.9459	0.0541	0.0000	Alt/Alt
NA19116	2	0	0.0000	0.3908	0.6092	Ref/Ref
NA19119	0	1	0.5223	0.4777	0.0000	Alt/Alt
NA19129	0	4	0.8974	0.1026	0.0000	Alt/Alt
NA19130	0	6	0.9722	0.0278	0.0000	Alt/Alt
NA19138	0	11	0.9991	0.0009	0.0000	Alt/Alt
NA19152	1	0	0.0001	0.5620	0.4380	Ref/Alt

Continued on next page

Table S4 – continued from previous page

Individual	Allele counts		Posterior probabilities			True genotypes
	Ref	Alt	Alt/Alt	Ref/Alt	Ref/Ref	
NA19172	0	5	0.9459	0.0541	0.0000	Alt/Alt
NA19204	0	1	0.5223	0.4777	0.0000	Alt/Alt
NA19207	0	2	0.6862	0.3138	0.0000	Alt/Alt
NA19209	4	1	0.0000	0.9988	0.0012	Ref/Alt
NA19222	1	0	0.0001	0.5620	0.4380	Ref/Alt
